# Chromosomal instability in *Streptomyces avermitilis*: major deletion in the central region and stable circularized chromosome

**DOI:** 10.1186/1471-2180-10-198

**Published:** 2010-07-26

**Authors:** Wei Chen, Fei He, Xiaojuan Zhang, Zhi Chen, Ying Wen, Jilun Li

**Affiliations:** 1 State Key Laboratories for Agro-biotechnology, College of Biological Sciences, China Agricultural University, Beijing 100193, PR China

## Abstract

**Background:**

The chromosome of *Streptomyces *has been shown to be unstable, frequently undergoing gross chromosomal rearrangements. However, the mechanisms underlying this phenomenon remain unclear, with previous studies focused on two chromosomal ends as targets for rearrangements. Here we investigated chromosomal instability of *Streptomyces avermitilis*, an important producer of avermectins, and characterized four gross chromosomal rearrangement events, including a major deletion in the central region. The present findings provide a valuable contribution to the mechanistic study of genetic instability in *Streptomyces*.

**Results:**

Thirty randomly-selected "bald" mutants derived from the wild-type strain all contained gross chromosomal rearrangements of various types. One of the bald mutants, SA1-8, had the same linear chromosomal structure as the high avermectin-producing mutant 76-9. Chromosomes of both strains displayed at least three independent chromosomal rearrangements, including chromosomal arm replacement to form new 88-kb terminal inverted repeats (TIRs), and two major deletions. One of the deletions eliminated the 36-kb central region of the chromosome, but surprisingly did not affect viability of the cells. The other deletion (74-kb) was internal to the right chromosomal arm. The chromosome of another bald mutant, SA1-6, was circularized with deletions at both ends. No obvious homology was found in all fusion sequences. Generational stability analysis showed that the chromosomal structure of SA1-8 and SA1-6 was stable.

**Conclusions:**

Various chromosomal rearrangements, including chromosomal arm replacement, interstitial deletions and chromosomal circularization, occurred in *S. avermitilis *by non-homologous recombination. The finding of an inner deletion involving in the central region of *S. avermitilis *chromosome suggests that the entire *Streptomyces *chromosome may be the target for rearrangements, which are not limited, as previously reported, to the two chromosomal ends.

## Background

*Streptomyces *are a genus of Gram-positive, filamentous soil bacteria, which display complex morphological differentiation and produce a broad range of bioactive secondary metabolites such as antibiotics, immunosuppressants and cholesterol-lowering agents. These bacteria thus provide an important natural source of commercial products for the pharmaceutical and agricultural industries [1]. The *Streptomyces *genome consists of an 8- to 9-Mb linear chromosome, characterized by terminal inverted repeats (TIRs) and a protein covalently attached to 5' end [2-4]. This chromosome is inherently unstable, and frequently undergoes gross chromosomal rearrangements spontaneously as well as under various mutagenic treatments [5,6], particularly in terminal regions where almost no essential genes reside. Gross chromosomal rearrangements include deletion, amplification, arm replacement, and circularization [7-16]. This chromosomal instability leads to genetic instability, which is ubiquitous among *Streptomyces*, and affects nearly all life functions, *e.g*., differentiation, secondary metabolism, and response to environmental changes [5]. The chromosomal instability is not attributable to the linear chromosomal structure, since some mutants with circular chromosomes display even higher frequency of genetic instability [7,17,18]. Theoretically, gross chromosomal rearrangements can arise through both homologous recombination and non-homologous recombination pathways. However, the mechanisms underlying these types of rearrangement in *Streptomyces *are poorly understood.

*Streptomyces avermitilis *produces avermectins (macrocyclic lactone derivatives with potent anthelmintic properties) which are widely used in agriculture, veterinary medicine, and human medicine [4,19]. Sequencing of the 9.02-Mb genome of *S. avermitilis *has been completed [4]. Comparative analysis with *S. coelicolor *A3(2) revealed that *S. avermitilis *has a highly conserved 6.5 Mb "core" internal region and two variable "auxiliary" telomeric regions: a 2.0 Mb left arm and a 0.5 Mb right arm. The TIRs are solely located within the first 174 nucleotides at both ends of the chromosome [20]. Genetic instability had been well studied in several other *Streptomyces *species [10-16,21-24]. *S. avermitilis*, although not yet systematically investigated in this regard, is clearly subjected to genetic instability as well, since it frequently generates "white" or "bald" mutants showing reduction or complete loss of avermectin productivity. Such genetic instability is a significant problem for the commercial use of *S. avermitilis *in the fermentation industry as well as basic research, and therefore a better understanding of the mechanisms involved is needed.

In the present work, we examined the genetic instability of *S. avermitilis *using a combined approach of pulsed-field gel electrophoresis (PFGE), Southern hybridization, PCR, and DNA sequencing. The chromosomal structures of two bald mutants, SA1-6 and SA1-8, derived from spontaneous chromosomal rearrangement of the wild-type strain, were characterized in detail. Major deletion in the central region of the *Streptomyces *chromosome was observed for the first time in SA1-8, and stable circularized chromosome was observed in SA1-6. Analysis of the fusion sequences showed that non-homologous recombination was involved in the chromosomal rearrangements, including arm replacement, deletions and circularization. Lastly, the chromosome of SA1-6 and SA1-8 remained stable after ten passages, whereas other mutants such as SA1-7 and SA3-1 underwent further chromosomal rearrangements.

## Results

### Chromosomal instability in S. avermitilis

After serial transfer (more than 6 passages) on solid YMS medium, spores of *S. avermitilis *were harvested and suspended in distilled water. The spore suspension was re-plated on solid YMS to observe the phenomenon of morphological instability. Normal gray colonies appeared together with "white" mutants (*i.e*., defective in the ability to form mature spores) and bald mutants in the progeny. The mutants arose with a high frequency of 2.4% from the wild-type strain, and an even higher frequency of 8.3% from 76-9, a high avermectin-producing mutant. Thirty bald mutants from the wild-type strain and 30 bald mutants from 76-9 were randomly isolated, solely on the basis of their stable aerial mycelia-defective phenotype. Flask fermentation experiments and subsequent HPLC analysis demonstrated that all of these bald mutants lost the ability to produce avermectins (data not shown).

**Figure 1:**
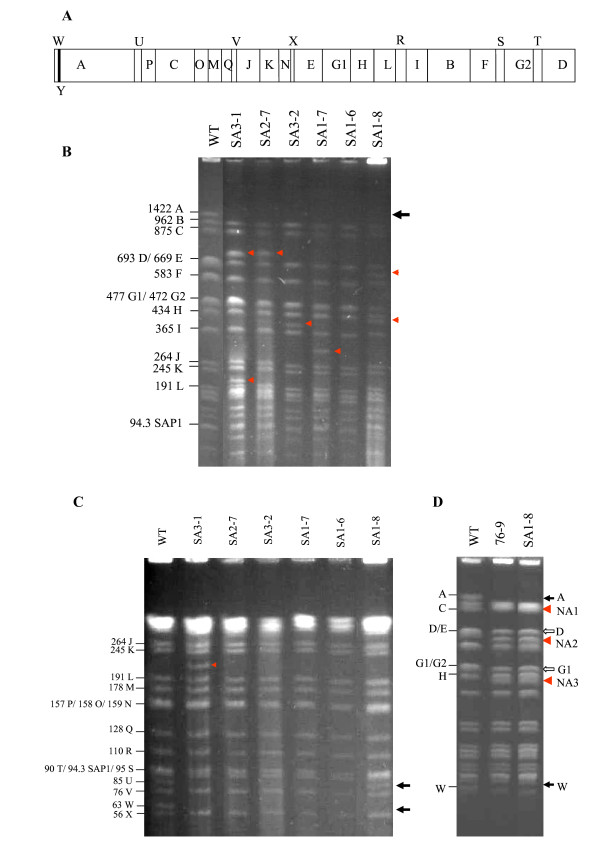
**Gross chromosomal rearrangements in spontaneous bald mutants from *S. avermitilis *wild-type (WT) strain ATCC31267**. (A) *Ase*I restriction map of wild-type chromosome. (B and C) *Ase*I restriction patterns of genomic DNA of bald mutants (SA). (D) Similar *Ase*I profiles of 76-9 and SA1-8. PFGE conditions for separating large fragments were: (B and D) 1.2% agarose, 4.5 V/cm, 20-130 s pulses, 36 h; 4.5 V/cm, 60-90 s pulses, 2 h; 4.5 V/cm, 5-10 s pulses, 8 h; conditions for separating small fragments were: (C) 1.5% agarose, 6 V/cm, 5-10 s pulses, 24 h. Fragments D and E overlapped because of their extremely similar migration; overlap was also found for fragments G1/G2, O/P/N, and S/T. SAP1: 94.3-kb linear plasmid. Solid arrows: missing fragments; Open arrows: potential missing fragments; Triangles: new bands.

To test whether chromosomes of the bald mutants were altered similarly to those in other *Streptomyces *species as reported previously [5], we conducted PFGE analysis of chromosomal structure. Through optimal adjustment of pulse time, 25 *Ase*I-fragments of *S. avermitilis *ATCC31267 (Fig. 1A) were successfully separated (except for 5-kb fragment Y) and varied in size from 57-kb to 1422-kb (Fig. 1B and 1C). Fragments D and W correspond to the right and left ends of the chromosome, respectively, which covalently bind terminal proteins. In comparison to *Ase*I patterns of wild-type chromosome, all the bald mutants derived from wild-type (designated SA) displayed chromosomal rearrangements. Some of the mutants shared similar PFGE profile representatively shown in Fig. 1B and 1C, although the chromosomal structures among these mutants might be different. Fragments *Ase*I-W (63-kb) and A (1422-kb) on the left chromosomal arm were involved in nearly all deletion events, most of which extended to fragment U (85-kb). Considering that the overlapping band D/E became fainter and thinner, it is most likely that the right terminal fragment D was missing, although the possibility that centrally located fragment E could also be missing can not be excluded. Meanwhile, some new *Ase*I bands appeared in the SA mutants. In contrast, the spontaneous bald mutants derived from 76-9 showed no apparent chromosomal rearrangements in comparison to the *Ase*I pattern of 76-9 (Additional file 1: Supplementary Fig. S1).

Among the rearrangement types of SA mutants, the *Ase*I profile of SA1-6 showed no novel bands apart from the deleted fragments (Fig. 1B and 1C). On the other hand, the *Ase*I profile of SA1-8 revealed two new fragments, and was quite similar to that of 76-9 (Fig. 1D), suggesting that SA1-8 and 76-9 may share the same chromosomal structure. Therefore, SA1-6 and SA1-8 were selected for further study of chromosomal architecture.

**Figure 2:**
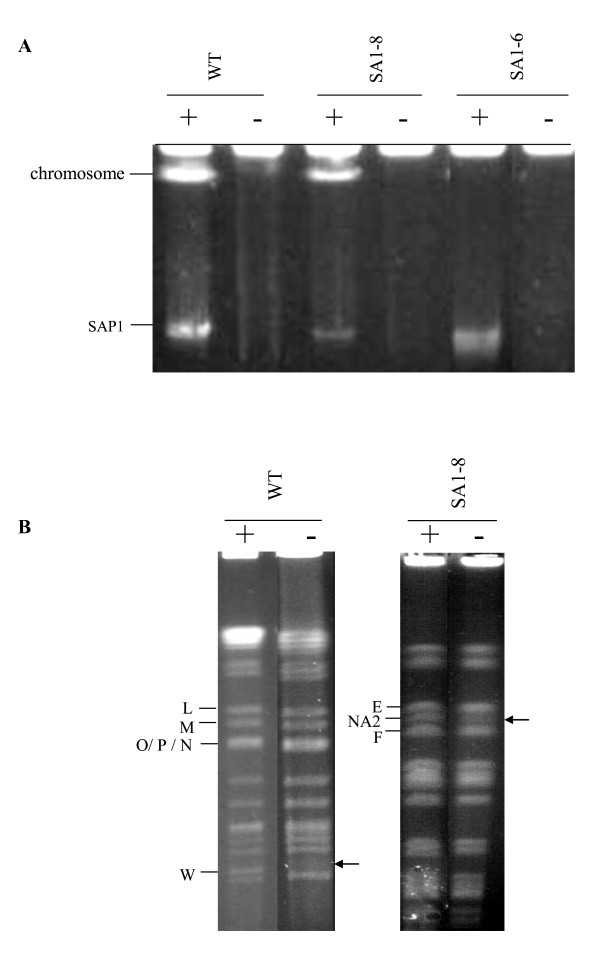
**PFGE analysis of the chromosomes of *S. avermitilis *strains**. (A) PFGE of intact chromosome treated with Proteinase K (PK) and SDS. (B) PFGE analysis of *Ase*I digested chromosome with PK and SDS treatment, showing that fragment NA2 is a new end bound to terminal protein. PFGE conditions for (A) were: 1% agarose, 3 V/cm, 180 s pulses, 20 h. Conditions for SA1-8 and wild-type in (B) were the same as for Fig 1B and 1C, respectively. "+" represents DNA sample treated with PK; "-'' represents DNA sample treated with SDS.

Both the linear chromosome and plasmid maintain a circular conformation *in vivo *because of the interaction of two terminal proteins. When intact DNA samples are treated with Proteinase K (PK), the covalently bound terminal proteins are removed and the DNA acquires a linear conformation. Whereas the intact DNA in the SDS-treated sample is trapped in the slot, since just noncovalently bound proteins are removed and the linear DAN keeps a circular form [3]. It has been reported that the wild-type strain ATCC31267 has a linear chromosome and a linear plasmid SAP1 of 94.3-kb [4]. When intact DNA samples of SA1-8, SA1-6 and the wild-type strain were electrophoresed under conditions allowing migration of only DNA linear structures, SA1-8 showed no detectable difference in banding pattern as compared to the wild-type strain, whereas only one plasmid band was detected in PK-treated DNA sample of SA1-6 (Fig. 2A), suggesting that the SA1-8 chromosome remained linear, whereas SA1-6 possessed a circular chromosome.

### Chromosomal arm replacement and internal deletions in SA1-8 chromosome

In comparison to the *Ase*I profile of wild-type, fragments W and A on the left chromosomal arm of SA1-8 were missing, and there were two novel fragments, which we termed NA2 and NA3 (Fig. 1D). To test whether the deletion of the W fragment included the left chromosomal terminus, we used probe W (754-1653 nt, relative to left first nucleotide of the chromosome defined as 1 nt) located on the left terminus, to hybridize onto the *Pst*I pattern of genomic DNA. The wild-type strain showed a predicted 1.6-kb restriction fragment, whereas SA1-8 showed no apparent hybridization signal (Additional file 1: Supplementary Fig. S2A), indicating that the left terminus was deleted. On the other hand, the right extremity was still conserved, since hybridization with probe Dr (196-bp away from the last nucleotide) showed that the terminal 4.7-kb *Bam*HI fragment was present in both wild-type and SA1-8 (Additional file 1: Supplementary Fig. S2B).

**Figure 3:**
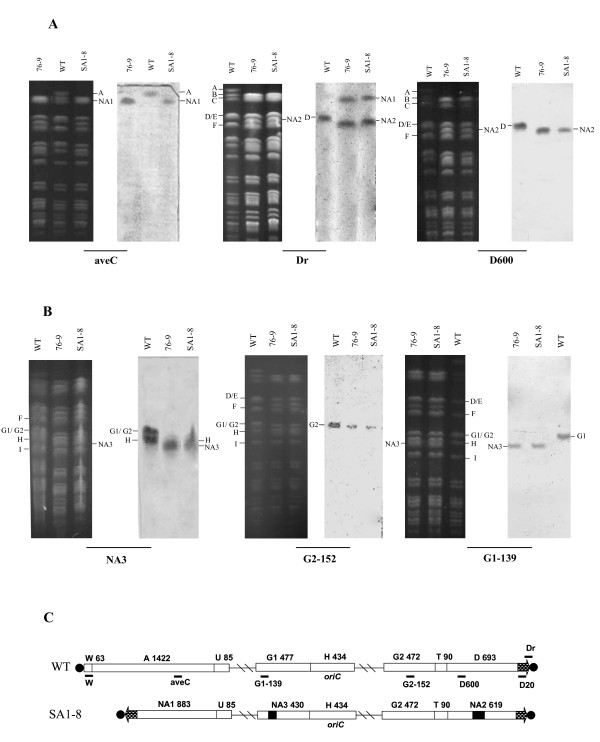
**Southern hybridization analysis of chromosomal rearrangements in SA1-8 (A, B) and schematic representation of the chromosomes of wild-type strain and mutant SA1-8, showing three independent rearrangements (C)**. Total DNAs were *in gel *digested with *Ase*I, and probed by aveC, Dr, D600, NA3, G2-152, and G1-139, respectively (A, B). Probe aveC was in the *ave *gene cluster of fragment A. Distance between probe and extreme right end of chromosome was 600-kb for D600, 196-bp for Dr. Probes G2-152 and G1-139 were located on fragments G2 and G1, respectively. PFGE conditions were the same as for Fig. 1B. (C) Open bar: simplified chromosome map with fragment designations and sizes in kilobases; Vertical lines: *Ase*I sites; Horizontal lines: probes; Diagonal lines: internal regions not displayed; Thick arrows: 88-kb TIRs; Solid circles: terminal proteins; Black bars: inner deletion regions.

Although SA1-8 lost the ability to produce avermecetins, the avermectin biosynthetic gene cluster, located within *Ase*I-A, could be specifically amplified by PCR (data not shown), indicating that fragment A was not deleted completely. To determine the remnant of fragment A, probe aveC (1,168,000-1,169,000 nt) in the *ave *gene cluster was amplified and labeled. Hybridization with this probe, surprisingly, revealed a new band (termed NA1) overlapping with fragment C (875-kb) (Fig. 1D and 3A). Fragment NA1 was also detected by the right terminal probe Dr, which hybridized with fragment D in wild-type (Fig. 3A). These results suggest that the right end replaced the left end and joined the undeleted part of *Ase*I-A to form the novel left terminal fragment NA1.

Probe Dr hybridized simultaneously with fragments NA1 and NA2 in SA1-8, and with fragment D in wild-type, suggesting that NA2 was derived from fragment D (Fig. 3A). Hybridization with probe D600 confirmed the loss of 693-kb *Ase*I-D, and formation of new ~600-kb NA2 in SA1-8 (Fig. 3A). When proteinase treatment was omitted, neither NA2 in SA1-8 nor *Ase*I-W in wild-type entered the PFGE gel (Fig. 2B). Slowing of fragment D in wild-type and of NA1 in SA1-8 could not be observed since they overlapped with fragments E and C, respectively. These findings indicate that reduction of fragment D led to the formation of NA2, which corresponds to the new right terminal end.

In order to determine the source of fragment NA3, the ~400-kb NA3 fragment was recovered with low-melt agarose and labeled. Hybridization studies of this probe with the PFGE-separated wild-type genomic *Ase*I fragments suggested that either G1 or G2 may be the source of NA3 (Fig. 3B), since G1 and G2 overlap. The NA3 probe also hybridized with fragment H of SA1-8 and wild-type, because H was close to NA3, and the recovered NA3 sample used for probe preparation was easily contaminated with DNA from H. Unstable regions are often localized at the telomere or subtelomere of the chromosome in *Streptomyces*, we therefore firstly attempted to identify the deletion in G2. Southern analysis with probe G2-152 showed that G2 remained intact (Fig. 3B), consistent with PCR results (data not shown). To test the possibility that central fragment G1 underwent deletion to form NA3, we performed hybridization using probe G1-139 located on G1. Probe G1-139 was found to hybridize with NA3 (Fig. 3B), suggesting that NA3 resulted from the reduction of G1.

### The extent of deletions and sequence of three junction fragments in SA1-8 chromosome

**Figure 4:**
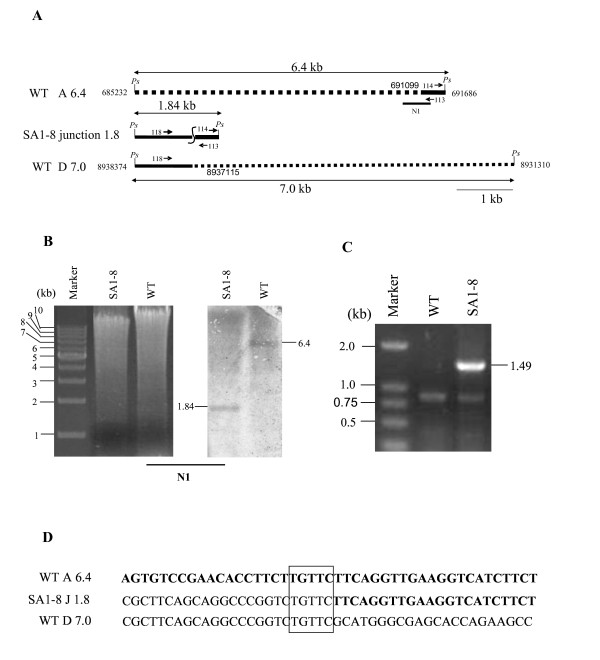
**Analysis of recombination point in fragment NA1**. (A) Restriction maps of fragments involved in the recombination event in NA1. The 1.84-kb *Pst*I junction fragment resulted from fusion in opposite orientation of partially deleted 6.4-kb and 7.0-kb *Pst*I fragments from left and right chromosomal arms, termed A6.4 and D7.0 respectively. (B) Hybridization analysis of the *Pst*I fusion fragment. (C) Inverse PCR to obtain the left unknown sequence of 1.84-kb *Pst*I junction fragment. (D) The fusion sequence in NA1 joins the partial region of fragment A6.4 and D7.0 at a 5-bp overlapping sequence. Bold and non-bold fonts represent nucleotide sequences from fragment A6.4 and D7.0, respectively. Dashed lines represent deleted regions. *Ps*: *Pst*I. Primers 113 and 114 were used in inverse PCR. Primers 118 and 113 were used in PCR for amplifying fusion sequence.

To determine the extent of the deletion, we conducted "walking PCR" strategy to detect the relevant region in SA1-8. The entire fragment W and left part of fragment A were missing, and the deletion terminus of fragment A was located near the 691200 nt locus. To confirm the breakpoint, we performed Southern analysis with probe N1 (690197-691592 nt, spanning the 691200 nt locus), which revealed a new 1.84-kb *Pst*I fragment in SA1-8, instead of the 6.4-kb *Pst*I fragment in the wild-type strain (Fig. 4A and 4B). The 1.49-kb fragment was obtained by inverse PCR using primers 113 and 114 (Fig. 4A and 4C). Sequence analysis revealed that the 1.49-kb fragment contained two parts, one from fragment D in the right chromosomal end, and the other from the remnant of fragment A. The junction sequence was further identified by PCR with primers 118 (located at *Ase*I-D) and 113 (located at *Ase*I-A) (Fig. 4A), using total DNA of SA1-8 as template. The breakpoint of fragment A was determined to be located at 691099 nt, with deletion of the left arm up to 691-kb, and fusion to 8937115 nt on the right chromosomal arm, 88-kb away from the extreme right end (Fig. 4A). Assuming that the entire right terminal 88-kb end translocated to the left breakpoint to form novel fragment NA1, the size of NA1 was estimated to be 882-kb (1422A+63W-691+88 = 882), which is consistent with the finding that NA1 co-migrated with fragment C (875-kb) in PFGE. This was further confirmed by results from Southern blotting, indicating that NA1 could hybridize with probes D20, D60, and D80 (20-, 60- and 80-kb away from the right extremity, respectively) (data not shown). Comparison of the junction sequence with the right and left sequences from the wild-type strain suggested that a non-homologous recombination event occurred within a short 5-bp region of homology (Fig. 4D).

**Figure 5:**
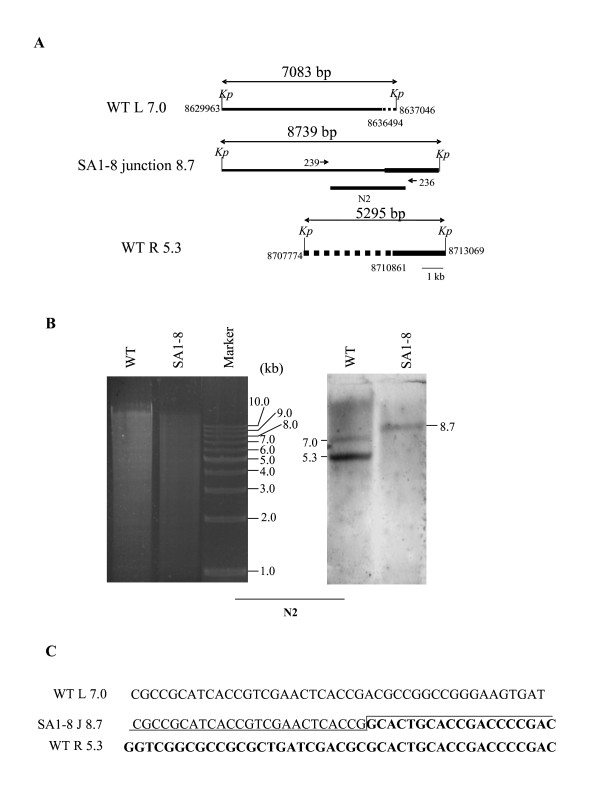
**Analysis of fusion sequence in fragment NA2**. (A) Location of chromosomal deletion ends and fusion junction. Left and right deletion termini were characterized by stepwise PCR mapping. Deleted and fused regions are indicated by dashed and shaded lines, respectively. *Kp*, *Kpn*I. (B) Southern analysis of fusion fragment with probe N2, which was prepared using primers 236 and 239. (C) Junction sequence, showing no obvious homology between the original sequences.

Walking PCR and sequence analysis showed that the left and right deletion termini in the interior of NA2 were located at 8636494 nt and 8710861 nt, respectively (Fig. 5A). The deletion extended to 74-kb, including 64 ORFs (SAV7241-SAV7304). The actual size of NA2 was therefore 619-kb (693D-74 = 619). These results also showed that the right terminal 88-kb fragment was conserved, since the right deletion termini was 314-kb away from the right extremity. We directly amplified and sequenced the newly formed DNA junction sequence with primers 236 and 239 flanking the fusion site. Breakpoint sequence analysis showed that the junction joined the partial regions of left 7.0-kb and right 5.3-kb *Kpn*I fragments, generating a new *Kpn*I fragment of 8.7-kb (Fig. 5A). This was confirmed by hybridization with probe N2 (Fig. 5B). No significant similarity was found when the junction sequence was compared with the left and right sequences from the wild-type strain (Fig. 5C).

**Figure 6:**
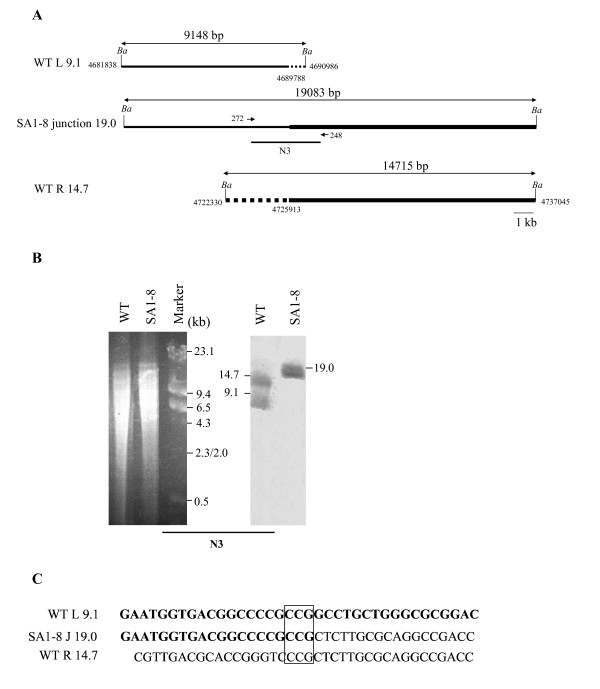
**Analysis of fusion sequence in fragment NA3**. (A) Location of chromosomal deletion ends and fusion junction. *Ba*, *Bam*HI. (B) Southern analysis of junction fragment with probe N3, which was prepared using primers 248 and 272. (C) Junction sequence in NA3. The 3-bp overlapping sequence is boxed.

The internal deletion region of G1 spanned from 4689788 nt to 4725913 nt, 562-kb away from the origin of replication (*oriC*). The results also suggested that the deletion terminated in the left 9.1-kb and right 14.7-kb *Bam*HI fragments, respectively, producing a novel 19.0-kb junction fragment (Fig. 6A). This was confirmed by Southern analysis using probe N3 (Fig. 6B). The fusion sequence acquired by direct PCR amplification with primers 272 and 248 suggested that a non-homologous recombination event had occurred, leading to loss of the intervening 36-kb DNA sequence (Fig. 6C). However, the reduction of G1 was estimated to be at least 43-kb (477G1-434H = 43), since NA3 was smaller than H (Fig. 1D). Another small size (~7-kb) deletion presumably occurred at an undetermined location within G1.

The deleted 36-kb region of G1 contained 32 ORFs from SAV3792 to SAV3823, including 14 hypothetical proteins. Since the substrate mycelia of SA1-8 could form normally, these genes are evidently not essential for growth of *S. avermitilis*. Among these ORFs, 13 genes (40%) had orthologs in *S. coelicolor *A3(2), and 12 genes (37%) were unique to *S. avermitilis*. The GC content of this region (70.5%) was not distinct from the average GC content of the *S. avermitilis *chromosome (70.7%). We did not find any transposable sequences or typical repeated sequences such as tRNA genes flanking the deleted region. It therefore seems unlikely that the deleted region was acquired from other species by horizontal gene transfer.

### Similar chromosomal structure of SA1-8 and 76-9

Based on the results described above, we are able to deduce the chromosomal structure of SA1-8, including at least three independent rearrangements: arm replacement, *i.e*., the 691-kb left end was deleted, and the 88-kb right terminal fragment was duplicated and translocated to the left end to form new 88-kb TIRs in SA 1-8, in place of the original 174-bp nucleotides in wild-type; the 36-kb deletion within central fragment G1; the 74-kb deletion within right terminal fragment D (Fig. 3C). Using corresponding primers, the same fusion sequences could be amplified from 76-9 as from SA1-8 (data not shown). Taken together, the PFGE patterns (Fig. 1D) and Southern hybridization results (Fig. 3A and 3B) indicated that 76-9 and SA1-8 have the same chromosomal structure, and have undergone the same three rearrangement events. Since 76-9 is able to sporulate and to produce high-level avermectins, it can be concluded that the deleted central region within G1 is not responsible for the differentiation or avermectin production in *S. avermitilis*.

### Chromosomal circularization in SA1-6

**Figure 7:**
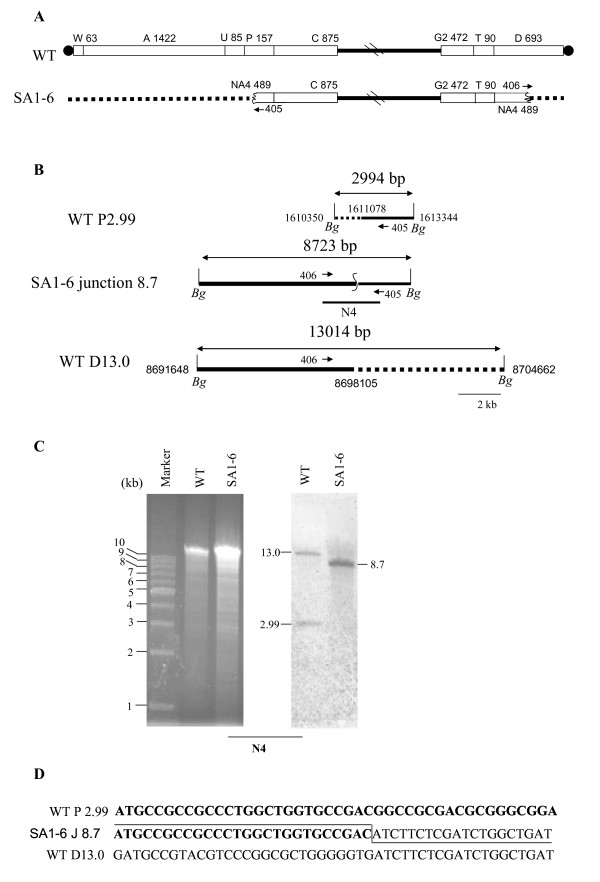
**Characterization of circular chromosome in SA1-6**. (A) Schematic representation of the chromosomes of wild-type strain and mutant SA1-6, showing deletions at both ends. (B) Location of chromosomal deletion ends and fusion junction. *Bg*, *Bgl*II. (C) Southern analysis of fusion fragment with probe N4, which was prepared using primers 405 and 406. (D) Junction sequence, showing no obvious homology between the original sequences.

The 1938-kb deletion region at both chromosomal ends of SA1-6 was identified by walking PCR, including entire *Ase*I-W, A, U, left part of *Ase*I-P, and right part of *Ase*I-D (Fig. 7A). No obvious retardation of the *Ase*I fragment of SA1-6 was observed in SDS-treated sample (data not shown), together with the intact chromosome remaining trapped in the gel well in PK-treated sample (Fig. 2A), indicating that the SA1-6 chromosome was circularized. The left and right deletion ends were located at 1611078 nt and 8698105 nt, respectively. Therefore, the size of the new *Ase*I junction fragment NA4 was 489-kb and overlapped with *Ase*I-G1 in the PFGE gel, which was confirmed by Southern hybridization using probe N4 spanning the fusion site (Additional file 1: Supplementary Fig. S3). Hybridization of probe N4 with the *Bgl*II-digested genomic DNA revealed that a 2.99-kb *Bgl*II fragment from the left *Ase*I-P and a 13.0-kb *Bgl*II fragment from the right *Ase*I-D in the wild-type strain were partially deleted and joined, generating a newly 8.7-kb *Bgl*II fragment in SA1-6 (Fig. 7B and 7C). No homology was found when the fusion sequence was compared with the corresponding left and right sequences from wild-type (Fig. 7D).

### Stability assay of chromosomal structure in bald mutants

Generational studies were performed to assess the chromosomal stability of bald mutants derived from the wild-type strain. Four bald strains were selected, and subjected to PFGE analysis following ten passages. The chromosomal structure of SA1-8 and SA1-6 was conserved, whereas that of SA1-7 and SA3-1 was changed (Additional file 1: Supplementary Fig. S4A). Both SA1-7 and SA3-1 lost their characteristic bands, and became indistinguishable from SA1-6. SA1-7 chromosome was further monitored in each passage, and found to change in the 4th passage (Additional file 1: Supplementary Fig. S4B). The corresponding fusion fragments of SA1-6 and SA1-8 were also detected in their progeny. These results indicate that chromosomal structure of SA1-6 and SA1-8 is stable.

## Discussion

This study demonstrated for the first time chromosomal rearrangement events in *S. avermitilis*, including chromosomal arm replacement, internal deletions and circularization. The chromosomal arm replacement in the bald mutant SA1-8 consisted of deletion of the 691-kb left terminus, and duplication of the 88-kb right terminus. The resulting new junction in fragment NA1 joined the partial coding regions of SAV546 (putative dehydrogenase) and SAV7499 (putative two-component system response regulator) at a 5-bp overlapping sequence. The internal deletions of fragments D and G1 appeared to be direct recombination events between two points. Fragment D was reduced 74-kb from SAV7241 to SAV7304. No significant homology was found, since the former was a putative ATP-dependent Clp protease, and the latter was a hypothetical protein. G1 had a 36-kb deletion, from SAV3792 to SAV3823, and the left and right deletion termini overlapped only by 3-bp nucleotides. The circular chromosome of SA1-6 joined SAV1302 (acetyl xylan esterase) and SAV7294 (amino acid transporter protein) with no overlapping sequence. Thus, all fusion sequences displayed minimal or no homology, indicating that the chromosome alteration has resulted from non-homologous recombination. Similarly, non-homologous (sometimes termed "illegitimate") recombination appeared to be involved in nearly all rearrangement events in previous studies of genetic instability in other *Streptomyces *species [5,9,12,14,21,25], except for two homologous recombinations occurring between duplicated genes [8,11]. This is reminiscent of breakpoint analysis of genome rearrangements in *Saccharomyces cerevisiae*, in which non-homologous end-joining (NHEJ) appeared to be the major mechanism involved in gross chromosomal rearrangements, even in those strains in which homologous recombination is functional [26]. Homologs of the eukaryotic DNA-end-binding repair protein Ku, involved in NHEJ pathway, have been found in *Streptomyces *[27], suggesting the presence of this pathway. It would thus be of interest to determine the relationship between Ku protein and chromosome instability in *ku *mutants of *Streptomyces*.

This is the first report of an inner deletion event involving the central region of the *Streptomyces *chromosome, suggesting that each part of the *Streptomyces *chromosome may be the target for rearrangements. Previous reports indicated that the two chromosome ends were primary targets for a variety of rearrangements: deletion, amplification, replacement, and circularization [5,9,14,25]. No essential genes located in the telomeric or subtelomeric regions of *Streptomyces *chromosome, and we are able to observe and characterize only those rearrangement events which did not affect the growth-dependent genes. This is the most likely reason as to why the majority of the rearrangements described in previous studies are located in the chromosome arms. The deleted central regions revealed in the present study are located between chromosomal region B (position 4,313,571-4,591,925 nt, SAV3480-SAV3709) and *oriC *(position 5,287,935- 5,289,024 nt) [20]. The 32 missing ORFs (Additional file 2) are unlikely to include any putative essential genes, since mutants SA1-8 and 76-9 both grew well on solid or in liquid medium. Similarly, Putnam *et al*. observed that any chromosomal region except centromeres in *S. cerevisiae *could be targeted by genome rearrangement, based on distribution of rearrangements in non-repetitive regions of the genome [26].

We found that the chromosomal structures of mutants SA1-8 and 76-9 were quite similar. The former resulted from spontaneous mutation of the wild-type strain, and the latter from various mutagenic treatments (UV, NTG, etc.). The phenotypes of SA1-8 and 76-9 were obviously distinct: SA1-8 was bald and did not produce avermectins, whereas 76-9 produced high level of avermectins and developed rich spores. Such differences presumably resulted from point mutations or small fragment changes involved in avermectin production and differentiation. On the other hand, some normal gray colonies of 76-9 underwent sequential differentiation into bald colonies, which remained the same chromosomal framework. This suggested that a chromosomal structure like that of 76-9 was relative stable. From a practical point of view, it would be valuable to complement such bald mutants with a gene library from 76-9 or the wild-type strain. If some mutation hot spots were identified and suppressed artificially, it would be possible to construct stable, high avermectin-producing strains. Such possibilities are being currently considered as part of ongoing studies in our laboratory.

Previous studies showed that artificially or naturally circularized chromosome of *Streptomyces *usually exhibited genetic instability similar to or at higher rates than the parent linear chromosome [7,17,18]. One possible explanation for the instability of circular chromosomes is lack of replication terminator structures or segregation elements, which are both necessary to maintain chromosome integrity [7]. However, two mutants, 404-23 and N2 from *S. griseus*, stably maintained their circular chromosomes [9], as was the case for mutant SA1-6 in the present work. It was postulated by Kameoka et al. that circularization prevented deletions from progressing into indispensable regions [9]. However, the regions near the deletion ends in SA1-6 don't contain any essential genes and thus the cause for stability of circular chromosomes in *Streptomyces *still remains to be elucidated.

Notably, we found that the essential chromosome structures of genetic instability mutants SA1-8 and SA1-6 were retained, whereas other dynamic mutants such as SA1-7 and SA3-1 underwent continuous chromosomal rearrangement. Similar phenomena were observed in *S. coelicolor *[14]. The mechanisms driving such gradual alterations of chromosomes are unclear. Alteration of an unstable monocentric chromosome in *S. cerevisiae *was attributed to joining of two "incompatible" regions [28]. In analogy, a plausible hypothesis in the present study is that the chromosomes of *S. avermitilis *mutants SA1-8 and SA1-6 were formed compatibly, whereas chromosomes of SA1-7 and SA3-1 harbored incompatible junction. However, what makes a stable junction "compatible", and what leads to "incompatibility" of two chromosome regions, remain to be clarified. Breakpoint analysis of the unstable chromosome of SA1-7 may shed some light on this issue.

The inherent chromosome instability of *Streptomyces *likely reflects an evolutionary strategy for adapting to environmental changes by creating populations with altered genetic information [29]. Unfortunately, this "strategy" often results in reduced production of secondary metabolites which are desired in agricultural, pharmaceutical, and research industries. From this point of view, the present findings contribute to elucidation of mechanisms underlying genetic instability in *Streptomyces*, and may help devising approaches to suppress or control such instability for industrial purposes.

## Conclusions

*S. avermitilis *underwent chromosomal rearrangement events, including chromosomal arm replacement, internal deletions and circulation, by non-homologous recombination. The fact that major deletion in the central region of chromosome was observed in *S. avermitilis *suggests that genetic instability of the *Streptomyces *chromosome is uniform across the entire chromosome. Stability assay showed that the chromosome of some bald mutants derived from the wild-type strain was conserved, whereas other mutants underwent further chromosomal rearrangement.

## Methods

### Bacterial strains and growth conditions

*S. avermitilis *ATCC31267 (wild-type strain) was used as starting strain and control. 76-9 was a high avermectin-producing strain derived from ATCC31267 by continuous mutagenesis, with the ability to sporulate. Spontaneous "bald" mutants (*i.e*., defective in production of aerial mycelia) of ATCC31267 and 76-9 were picked at random for further study, since the bald phenotype was stable. All strains were grown at 28°C on YMS solid medium for sporulation [30], or for isolation and growth of bald colonies.

### Preparation of DNA for PFGE analysis

*S. avermitilis *was cultured at 28°C for 36 h in 25 mL YEME with 25% sucrose in a 250 mL flask, containing a coiled stainless steel spring to promote aeration and cell dispersion. Mycelia were harvested and used for making plugs, as described by Kieser *et al *[31]. For restriction analysis, 200 μl buffer (per manufacturer's instructions) was added into 1.5 mL eppendorf tube containing one plug, incubated for 30 min at room temperature, and then the buffer was replaced with 300 μl fresh buffer containing 2 μl BSA (100 μg/mL) and 50 U *Ase*I to digest the plug for 4 h at 37°C. PFGE runs were performed in a CHEF MAPPER XA system (Bio-Rad). Agarose gels were run in 0.5 × TBE buffer at 14°C. Pulse times were optimized depending on the sizes of the DNA fragments to be separated. Proteinase K or SDS-treated DNA samples were prepared as described by Kieser *et al *[31].

### Detection of chromosomal deletion by PCR amplification

Using the available genomic sequence of *S. avermitilis *http://avermitilis.ls.kitasato-u.ac.jp, PCR primers were designed to detect chromosomal deletion, with DNA extracted from the wild-type strain as positive control.

### Inverse PCR

To determine the fusion sequence in novel fragment *Ase*I-NA1 of mutant strain SA1-8, inverse PCR was conducted. Total DNA of strain SA1-8 was completely digested by *Pst*I, and separated by conventional agarose gel electrophoresis. Fragments from 1-3 kb were retrieved, purified, and self-ligated. Circular DNA was used as template for inverse PCR. Since walking PCR and Southern blotting analysis had revealed the formation of a new ~2 kb *Pst*I fragment spanning the breakpoint of NA1 and the sequence of the right part of this new *Pst*I fragment (see the results), two primers, 113 (GGACTACGCCTTCGACTTC) and 114 (GATCGTGTACTGGGACCAG) in the right known region of the new *Pst*I fragment, were designed in opposite directions to amplify the left unknown sequence. Primer 113 was close to the breakpoint and primer 114 was near the right *Pst*I site. After sequencing analysis of the inverse PCR product, primers 118 (GTATCTCTCGTACGCCTCG) and 113 were used to determine the junction sequence (Fig. 4A).

### DNA labeling and hybridization

Following PFGE or conventional agarose gel electrophoresis, DNA fragments were transferred to nylon filters (Hybond-N, Millipore) by capillary method [32], and cross-linked by exposure to 254 nm UV for 10 min. DNA probes for Southern blotting were amplified by PCR, and labeled using a nonradioactive digoxigenin (DIG) labeling kit (Roche). Hybridization and detection were performed according to the manufacturer's instructions. Primers used for preparation of probes are listed in Table 1.

**Table 1:** Primers used for preparation of probes

Probes	Primers sequence (5'-3')		Description
W	11	GTTGCGGACGTGTGACTTG	to detect the left extremity of chromosome
	12	GAACTACATGCCGGGAGTG	

aveC	15	CAGCAAGGATACGGGGAC	to detect the avermectin biosynthetic gene cluster
	16	ACCGAGCACGATGCCGATG	

G1-139	139	GGTTGACGACGTCCTTGAG	to detect fragment *Ase*I-G1
	140	CGACACTCATGAAGCGACC	

G2-152	152	CTGCTCAAGACGAAGGTGC	to detect fragment *Ase*I-G2
	153	CCGTCACATCGCTGTCATG	

D600	131	GTGATCGTGAAGACCTCGC	to detect fragment *Ase*I-D
	132	CTCCACCATGACAAGACCG	

Dr	19	AGTCGTACGTCCGCAACTG	to detect the right extremity of chromosome
	20	AGGTCTTCCGCTTCGCTTC	

D20	125	CTCTAGACGGCGGAATCAC	to detect the duplication and translocation of right 88-kb end
	126	GCGACAAGGGCTAAGACTC	

D60	96	GTTCTGGCAGTCGTCGTAG	to detect the duplication and translocation of right 88-kb end
	97	TGAAGAAGACCCGGTCTGG	

D80	98	ACGAACGTGCCCTGCTCAC	to detect the duplication and translocation of right 88-kb end
	99	GGTGACGAGTTCGGAGACG	

N1	75	GGAGGTAGCGGATGTTGTG	to detect the rearrangement event in fragment *Ase*I-NA1
	76	CTGGTCCCAGTACACGATC	

N2	236	GGCTCGTTCATCTTCCTCG	to detect the rearrangement event in fragment *Ase*I-NA2
	239	GCACATCAGAGGGTCATGC	

N3	272	CGTTGACGTAGAGCTGCG	to detect the rearrangement event in fragment *Ase*I-NA3
	248	ACCTGAGCAGCTCGTGAAG	

N4	405	TGTGACGGTGTGCCAGTAG	to detect the rearrangement event in fragment *Ase*I-NA4
	406	ATGCCCTCGACTACGACAC	

### Chromosomal stability assay of mutants

To assess chromosomal stability of mutants over generations, selected bald mutants derived from wild-type were grown for ten passages on YMS plates. Since aerial mycelia of *S. avermitilis *begin to emerge after 48 h of incubation on YMS, we transferred mycelia of bald mutants grown for 3 days by streaking on YMS plates. Genomic DNA was analyzed by PFGE as described above.

## Authors' contributions

WC carried out most of the experiments and wrote the draft manuscript. FH and XZ performed some research on characterizing the circular chromosome of mutant SA1-6. ZC assisted with experimental design and data analysis. YW and JL supervised the whole work and revised the manuscript. All authors read and approved the final manuscript.

## Supplementary Material

Additional file 1 **Supplementary Fig. S1**. *Ase*I restriction patterns of genomic DNA of spontaneous bald mutants from 76-9. **Supplementary Fig. S2**. Southern hybridization analysis of the left (A) and right end (B) of the SA1-8 chromosome. **Supplementary Fig. S3**. Southern hybridization analysis of *Ase*I macrorestriction fragments of the SA1-6 chromosome with probe N4. **Supplementary Fig. S4**. Generational stability analysis of bald mutants. Click here for file

Additional file 2 **Complete data for deletion extent of fragment G1**. Click here for file
