# Supplementary material for: Chromosomal instability in Streptomyces avermitilis: major deletion in the central region and stable circularized chromosome
Source: BMC Microbiol. 2010 Jul 26;10:198. doi: 10.1186/1471-2180-10-198 (PMC2920896; doi:10.1186/1471-2180-10-198)
Supplement: Additional file 1 — Supplementary Fig. S1. AseI restriction patterns of genomic DNA of spontaneous bald mutants from 76-9. Supplementary Fig. S2. Southern hybridization analysis of the left (A) and right end (B) of the SA1-8 chromosome. Supplementary Fig. S3. Southern hybridization analysis of AseI macrorestriction fragments of the SA1-6 chromosome with probe N4. Supplementary Fig. S4. Generational stability analysis of bald mutants. [file 1471-2180-10-198-S1.PDF]

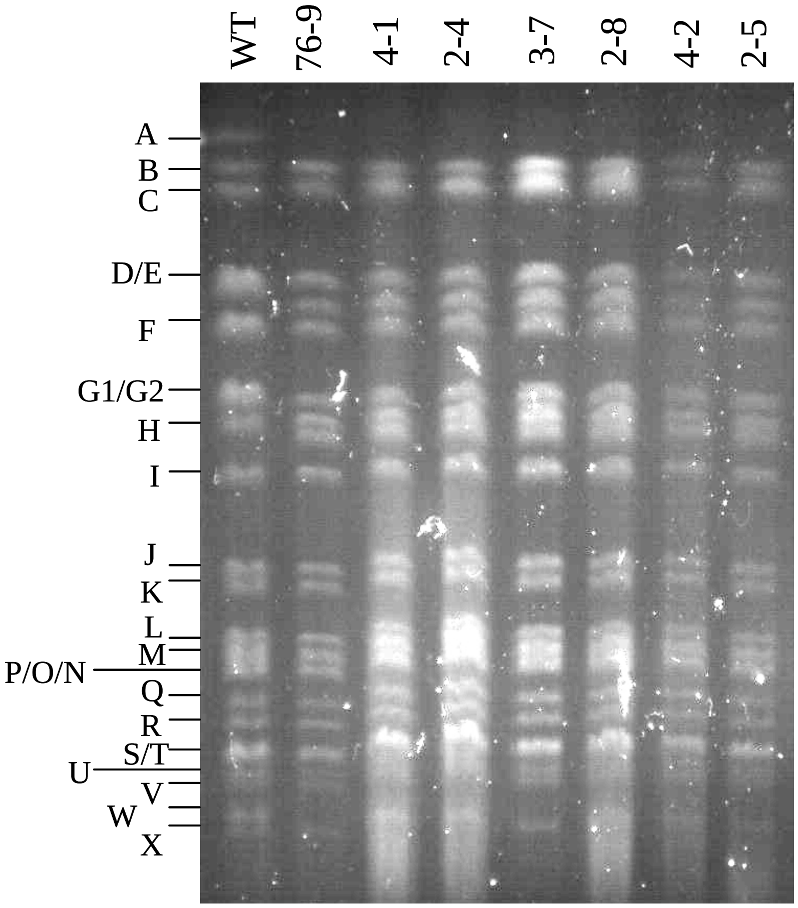

Fig. S1. *Ase*I restriction patterns of genomic DNA of spontaneous bald mutants from 76-9. No obvious rearrangements were found among derivatives of 76-9.

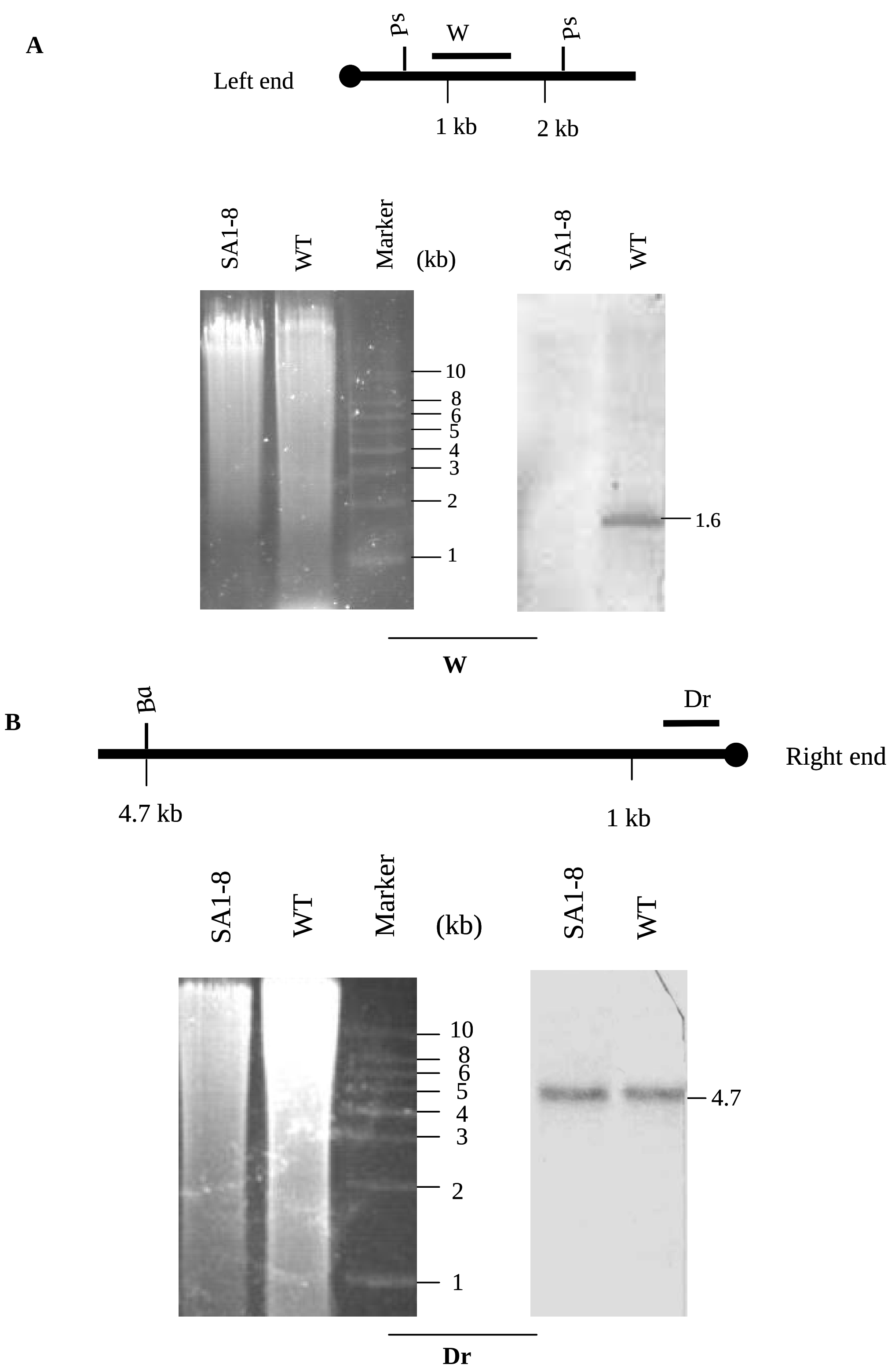

Fig. S2. Southern hybridization analysis of the left (A) and right end (B) of the SA1-8 chromosome. Only *Pst*I (*Ps*) and *Bam*HI (*Ba*) sites are shown for the left and right end, respectively. Locations of probe W and probe Dr are indicated.

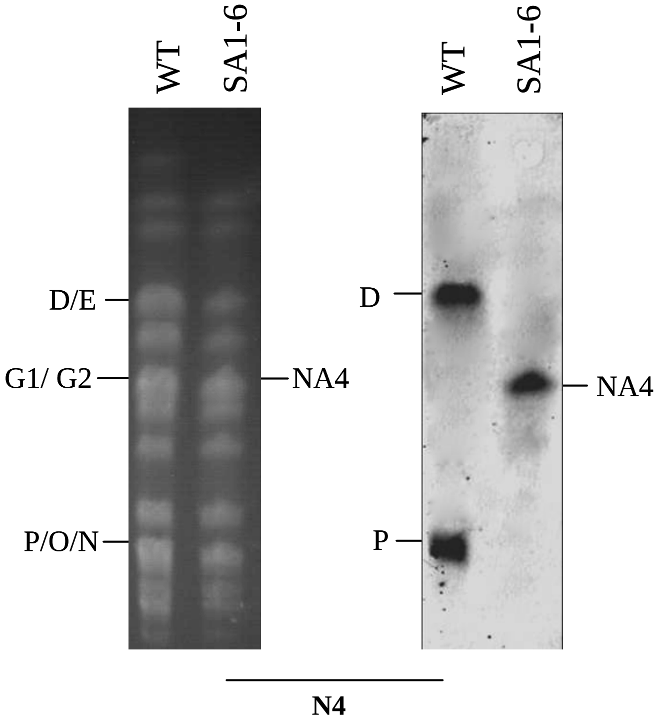

Fig. S3. Southern hybridization analysis of *Ase*I macrorestriction fragments of the SA1-6 chromosome with probe N4, which detected the novel junction fragment NA4 formed by joining the partial deleted fragment P and fragment D.

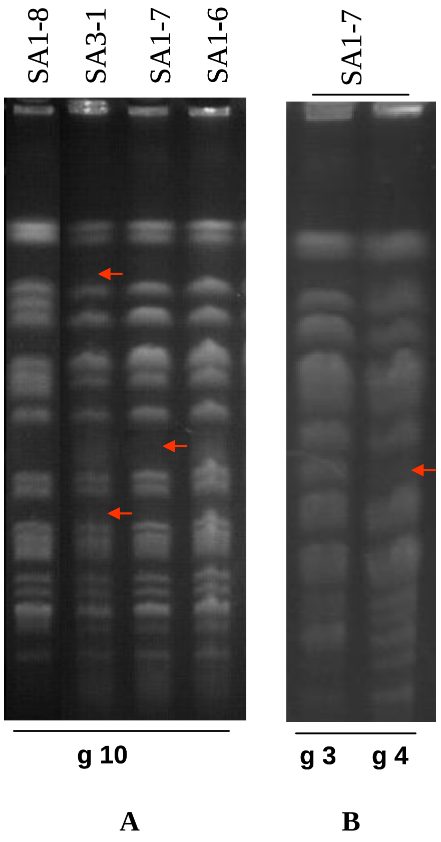

Fig. S4. Generational stability analysis of bald mutants. (A) Chromosomal structures of SA1-8 and SA1-6 remained unchanged after ten passages of growth, whereas those of SA1-7 and SA3-1 were altered. Characteristic novel bands of SA1-7 and SA3-1 were lost, without appearance of new bands. (B) The chromosome of SA1-7 changed in the 4th passage. Arrows: missing bands.
